# Time series of hospitalizations for primary care-sensitive conditions in children in the state of Roraima, Brazil, 2010 to 2023

**DOI:** 10.1590/1980-549720250016

**Published:** 2025-04-07

**Authors:** Ellen Vanuza Martins Bertelli, Gislayne Cristina Torreias de Carvalho, Raphael Mendonça Guimarães, Viviane Gomes Parreira Dutra

**Affiliations:** I Universidade Estácio de Sá - Rio de Janeiro (RJ), Brazil.; II Universidade Estadual de Roraima - Boa Vista (RR), Brazil.; III Fundação Oswaldo Cruz - Rio de Janeiro (RJ), Brazil.

**Keywords:** Primary health care, Child health, Hospitalization, Epidemiology

## Abstract

**Objective:**

To analyze the trend of hospitalizations for primary care-sensitive conditions in children in the state of Roraima, Brazil.

**Methods:**

Ecological time series study with secondary data collected from the Hospital Information System, on hospitalizations for primary care-sensitive conditions in children between 2010 and 2023.

**Results:**

The highest hospitalization rates were in 2021 (265.9/10,000 inhabitants/year) and the under 1 age group had the highest rates. There was a reduction in ambulatory care-sensitive conditions in group 1, in the first and second segments (MPC=-1.50; 95%CI -3.79-0.32 p=0.016 and MPC=-1.91; 95%CI -3.44--0.81 and p=0.007), group 2, with a constant drop throughout the series of 0.57% per month (95%CI -0.70--0.43 and p<0.001) and in group 16 (MPC=-0.38; 95%CI -0.55--0.21 and p<0.001). Bacterial pneumonia was the main cause of hospitalization in all age groups. Group 4 showed a uniform increase in rates of 1.56% per month (95%CI 0.27-2.80 and p=0.018). There was a drop in rates in the first segment at all ages: <1 year: -2.90% and p=0.019; 1 to 4 years: -1.75% (p=0.011) and 5 to 9 years: -0.79% (p=0.053), followed by an increase and then a drop again. In the last segment, all the age groups showed an increase in rates.

**Conclusions:**

The scenario in Roraima is worrisome and requires urgent interventions, as there is evidence of weaknesses in primary health care, probably aggravated by the migratory crisis. The strategies sought are still insufficient.

## INTRODUCTION

Primary health care (PHC) coordinates health care^1^, which means promoting integration between actions, services and professionals, using specific mechanisms and instruments for planning care, defining flows, and exchanging information with users and among professionals. Therefore, the implementation of indicators that monitor and evaluate the effectiveness of PHC is essential, as such indicators provide an objective measure of the performance of the care offered to users and allow for systematic evaluation^1^ ^,^ ^2^.

The health indicators for the Northern Region of Brazil highlight the weaknesses and health inequities that exist there^3^. In 2022, the North had an infant mortality rate of 15.1/1,000 live births, above the national rate of 12.6/1,000, revealing itself as the worst rate among the regions. The mortality rate for children under 5 years of age was 18.5/1,000 live births, while in Brazil it was 15/1,000. The maternal mortality rate in the region was also the highest in Brazil, reaching 74.5/100,000 live births^4^.

Among the states in the region, Roraima has the smallest population, with 636,303 inhabitants, and the fewest municipalities, just 15^5^. Although it is the smallest state in the country, it has been facing significant challenges in the organization of health services for some time. On the one hand, there is an intense flow of migrants, predominantly of Venezuelan origin, due to a humanitarian crisis in that country^6^. In addition, there is an intensification of territorial conflicts involving indigenous populations, who have been isolated and, consequently, experience barriers to accessing any health service^7^. An example of this was the measles outbreak in 2018, which exposed the state’s inefficient vaccination coverage, which was less than 95%^8^.

The state’s health system faces significant challenges, mainly associated with increasing family health coverage and expanding hospital beds below the national average^9^. According to data from Giovanella et al.^10^, we found that the coverage of the Family Health Strategy (FHS) is insufficient, with rates below the national average. The population coverage by the FHS in Roraima is 48.75%, according to data from May 2023 from the AB e-Manager of the Ministry of Health, versus 62.1% of national coverage. Regarding the density of doctors, the state has 1.82 doctors per 1,000 inhabitants, which is equivalent to 18.2 doctors for every 10,000 inhabitants, compared to 24/10,000 in the country as a whole. However, 97% of these professionals are in the capital, Boa Vista, resulting in an unequal distribution. Regarding hospital beds, Roraima showed a growth of 24.1% in the last decade, going from 1.73 to 2.15 beds per 1,000 inhabitants between 2012 and 2022. This corresponds to 21.5 beds for every 10,000 inhabitants.

One way to indirectly monitor the quality of primary care services is through the use of the indicator of hospitalizations for primary care-sensitive conditions (HPCSC). This indicator is commonly used worldwide as an indirect evaluator of the quality of PHC and has proven to be a reliable assessment parameter^11^. When PHC does not provide sufficient or adequate access to health services, there is an excessive demand for care at the secondary and tertiary levels, resulting in increased hospitalizations, costs and unnecessary travel^12^. In Brazil, Ordinance No. 221 of April 17, 2008^13^ defines the conditions considered preventable by PHC.

National studies on HPCSC in children indicates distinct epidemiological profiles in Brazilian states. In the recent literature, the only study that explores a state in northern Brazil is that of Santos et al.^14^, which analyzed Rondônia from 2008 to 2019 and identified that the majority of HPCSC in children were caused by skin infections or gastroenteritis. Regarding HPCSC in children in the state of Roraima, no studies were found that specifically addressed this topic. Therefore, this study aimed to analyze the trend of HPCSC in children in the state of Roraima.

### Study design and location

Ecological time series study conducted in Roraima. The state is located in the Northern Region of Brazil and is the least populous in the country, with 636,303 inhabitants, distributed across 15 municipalities, according to the 2022 census^15^. The population is composed of a notable presence of indigenous communities, representing 14.1% of the inhabitants, in addition to migrants, especially Venezuelans in recent years. Geographically, Roraima is characterized by extensive tropical forests and isolated communities, which makes logistics and the provision of essential services difficult. Furthermore, in recent history, the state has experienced migratory flows and disease outbreaks^16^.

### Data source

The data used were collected from the Hospital Information System of the Unified Health System (SIH-SUS, https://datasus.saude.gov.br/transferencia-de-arquivos/) and extracted from the website of the SUS Information Technology Department (DATASUS, https://datasus.saude.gov.br/), on HPCSC that occurred in children under 10 years of age between January 2010 and July 2023, in the state of Roraima. The set corresponds to all hospitalizations processed in the period and that were included in the hospitalization authorization, in the hospitalization justification field, one of the codes from the Tenth Revision of the International Statistical Classification of Diseases and Related Health Problems that are included in Ordinance No. 221, of April 17, 2008^13^, as per Chart 1.

**Chart 1:** Brazilian list of primary care-sensitive conditions, Brazil, 2008.

Group	Name	International Classification of Diseases, 10th Revision (ICD-10)
1	Vaccine-preventable and sensitive conditions diseases	A37, A36, A33 to A35, B26, B06, B05, A95, B16, G00.0, A17.0, A19, A15.0 to A15.3, A16.0 to A16.2, A15.4 to A15.9, A16.3 to A16.9, A17.1 to A17.9, A18, I00 to I02, A51 to A53, B50 to B54, B77
2	Infectious gastroenteritis and complications	E86 and A00 to A09
3	Anemia	D50
4	Nutritional deficiencies	E40 to E46, E50 to E64
5	Ear, nose and throat infections	H66, J00, J01, J02, J03, J06, J31
6	Bacterial pneumonias	J13, J14, J15.3, J15.4, J15.8, J15.9, J18.1
7	Asthma	J45, J46
8	Lung diseases	J20, J21, J40, J41, J42, J43, J47, J44
9	Hypertension	I10, I11
10	Angina	I20
11	Heart failure	I50, J81
12	Cerebrovascular disease	I63 to I67; I69, G45 to G46
13	Diabetes mellitus	E10.0, E10.1, E11.0, E11.1, E12.0,E12.1; E13.0, E13.1; E14.0, E14.1, E10.2 to E10.8, E11.2 to E11.8; E12.2 to E12.8; E13.2 to E13.8; E14.2 to E14.8, E10.9, E11.9; E12.9, E13.9; E14.9
14	Epilepsy	G40, G41
15	Kidney and urinary tract injections	N10, N11, N12, N30, N34, N39.0
16	Skin and subcutaneous tissue infections	A46, L01, L02, L03, L04, L08
17	Inflammatory disease of the female pelvic organs	N70, N71, N72, N73, N75, N76
18	Gastrointestinal ulcers	K25 to K28, K92.0, K92.1, K92.2
19	Prenatal and childbirth-related illnesses	O23, A50, P35.0

Source: Brasil^13^.

### Data analysis

Hospitalizations were analyzed by age group: under 1 year old, 1 to 4 years old and 5 to 9 years old, and by the groups defined in Ordinance No. 221/2008. To calculate hospitalization rates, we used as the denominator the annual population estimates from the Brazilian Institute of Geography and Statistics, available on the DATASUS website. Hospitalization rates for ACSC were calculated separately for each age group, using Equation 1:

number of HPCSC occurring per year and age group/population of the same year and in the same age group×10,000 (1)

We performed analysis for specific HPCSC groups, among the 19 groups existing in Ordinance No. 221/2008. It is important to highlight that groups whose outcomes were consistent with the child’s health were selected. The selected groups were:

- 1: Diseases preventable by immunization and sensitive conditions;
- 2: Infectious gastroenteritis and complications;
- 4: Nutritional deficiencies;
- 6: Bacterial pneumonia;
- 7: Asthma;
- 8: Lung diseases;
- 16: Infection of the skin and subcutaneous tissue;
- 19: Prenatal and childbirth-related illnesses.

The list of groups was validated by a panel of experts. In the analyses by age group, we used all the HPCSC that occurred in the period.

The HPCSC rate was considered the dependent variable, while the year was the independent variable. A segmented linear regression analysis was performed using the joinpoint method, which allowed us to identify the monthly increase, defined as the monthly percentage variation in the coefficients of hospitalizations due to PCSC. The method used adjusts the data of the series according to the smallest possible number of joinpoints (0, i.e., a straight line without inflection points) and tests whether the inclusion of more joinpoints is statistically significant. The significance tests applied were based on the Monte Carlo permutation method and on the calculation of the monthly percentage change of the ratio, using the logarithm of the ratio^17^. A 95% confidence interval (95%CI) was considered. The analyses were performed using the Joinpoint Regression Program, version 5.1.0.0.

The study followed the standards for research involving human beings, according to Resolution No. 466/2012. As this was a study conducted with secondary data, the source of which is public and the database is unidentified, the study was exempted from assessment by the Human Research Ethics Committee.

## RESULTS

**Figure 1:**
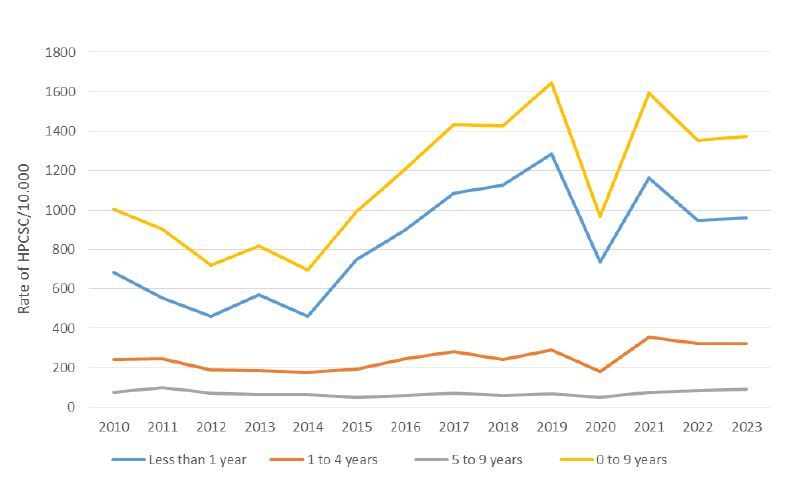
Rates of HPCSC/10,000 inhabitants by age group in children from 2010 to 2023. Roraima, Brazil.

During the study period, 35,684 cases of HPCSC were recorded in children under 10 years of age in the state of Roraima. Rates varied over the years and between different age groups. Overall, HPCSC rates showed a dynamic behavior, with a reduction in certain groups of causes and time periods, followed by growth in other periods (Figure 1).

HPCSC: hospitalizations for primary care-sensitive conditions.

Table 1 presents the analysis of HPCSC rates in different age groups, segmented by time periods. For each segment, monthly percentage change (MPC) values, 95%CI and statistical significance values (p value) are provided. The results showed distinct patterns between the age groups and time periods analyzed. In children under 1 year of age and children aged 1 to 4 years, recent periods showed a significant increase in HPCSC rates, especially after 2020. In the age group of 5 to 9, although growth was more modest, a significant increase was also recorded in the period from April 2020 to July 2023.

**Table 1:** Temporal trends of HPCSC rates according to cutoff points obtained through joinpoint by age group. Roraima, Brazil. January/2010 to August/2023.

Age group (years)	Segment/Period		MPC	95%CI		p value
				LL	UL	
<1	Jan/2010	Mar/2012	-2.90	-7.75	-0.64	0.019
	Mar/2012	Sep/2019	1.83	1.47	2.42	0.016
	Sep/2019	May/2020	-9.90	-30.59	-0.80	0.027
	May/2020	Jul/2023	2.61	1.33	5.32	0.007
	Jan/2010	Jul/2023	0.62	0.34	0.93	0.005
1 to 4	Jan/2010	Dec/2012	-1.75	-4.09	-0.51	0.011
	Dec/2012	Nov/2019	1.10	0.77	1.77	0.003
	Nov/2019	May/2020	-13.04	-30.24	-1.82	0.009
	May/2020	Jul/2023	4.43	3.38	5.92	0.001
	Jan/2010	Jul/2023	0.68	0.45	0.90	<0.001
5 to 9	Jan/2010	Apr/2015	-0.79	-2.30	-0.11	0.053
	Apr/2015	Jan/2020	0.47	-11.62	18.39	0.154
	Jan/2020	Apr/2020	-18.05	-25.66	11.53	0.128
	Apr/2020	Jul/2023	3.69	2.24	5.69	0.033
	Jan/2010	Jul/2023	0.36	0.15	0.59	0.019

HPCSC: hospitalizations for primary care-sensitive conditions; MPC: monthly percentage change; 95%CI: 95% confidence interval; LL: lower limit; UL: upper limit.

Among children under 1 year of age, rates decreased by 2.90% from January 2010 to March 2012 (p=0.019), followed by an increase of 1.83% until September 2019 (p = 0.016). Between September 2019 and May 2020, there was a decrease of 9.90% per month (p=0.027), with a subsequent increase of 2.61% until July 2023 (p=0.007). In the total period, there was an overall increase of 0.62% per month (p=0.005). In the age group from 1 to 4 years, there was a reduction of 1.75% per month from January 2010 to December 2012 (p=0.011), followed by an increase of 1.10% until November 2019 (p=0.003). From November 2019 to May 2020, there was a decrease of -13.04% per month (p=0.009), with a subsequent increase of 4.43% until July 2023 (p=0.001). In total, the increase was 0.68% per month (p<0.001).

Between 5 and 9 years, there was a reduction of 0.79% per month until April 2015 (p=0.053), an increase of 0.47% until January 2020 (not significant) and a decrease of 18.05% until April 2020 (not significant). From April 2020 to July 2023, there was a significant increase of 3.69% per month (p=0.033), with an overall increase of 0.36% in the period (p=0.019).

The HPCSC were analyzed in different groups of causes (PCSC), revealing distinct patterns of MPC throughout the periods (Table 2).

**Table 2:** Temporal trends of HPCSC rates according to cutoff points obtained through joinpoint by age group. Roraima, Brazil. January/2010 to July/2023.

PCSC group	Period/segment		MPC	95%CI		p value
				LL	UL	
Group 1	Jan/2010	Oct/2014	-1.50	-3.79	-0.32	0.016
	Oct/2014	Jun/2018	2.83	1.22	50.94	0.009
	Jun/2018	Jul/2023	-1.91	-3.44	-0.81	0.007
Group 2	Jan/2010	Jul/2023	-0.57	-0.70	-0.43	<0.001
Group 4	Jan/2010	Jul/2023	1.56	0.27	2.80	0.018
Group 6	Jan/2010	Nov/2012	-22.84	-31.19	-14.67	<0.001
	Nov/2012	Aug/2015	53.15	38.83	86.15	<0.001
	Aug/2015	Jul/2023	0.65	-1.59	2.69	0.567
Group 7	Jan/2010	Oct/2020	-1.45	-3.07	-0.31	0.036
	Oct/2020	Jul/2023	8.70	-0.49	53.27	0.098
Group 8	Jan/2010	Jul/2023	1.95	1.17	2.76	<0.001
Group 16	Jan/2010	Jul/2023	-0.38	-0.55	-0.21	<0.001
Group 19	Jan/2010	Feb/2011	-26.76	-89.89	3.67	0.178
	Feb/2011	Jul/2023	3.44	-97.43	6.31	0.080

HPCSC: hospitalizations for primary care-sensitive conditions; PCSC: primary care-sensitive conditions; MPC: monthly percentage change; 95%CI: 95% confidence interval; LL: lower limit; UL: upper limit.

Regarding infectious or vaccine-preventable diseases, we highlight that in group 1 (vaccine-preventable diseases and sensitive conditions), there was a reduction of 1.50% per month between January 2010 and October 2014 (p=0.016), followed by an increase of 2.83% per month until June 2018 (p=0.009) and a new reduction of 1.91% per month until July 2023 (p=0.007). In group 2 (infectious gastroenteritis), a constant reduction of 0.57% per month was identified between 2010 and 2023 (p<0.001). For group 4 (nutritional deficiencies), there was an increase of 1.56% per month in the same period (p=0.018). In group 6 (bacterial pneumonia), there was a reduction of 22.84% per month between 2010 and 2012 (p<0.001), an increase of 53.15% per month until 2015 (p<0.001) and a variation of 0.65% per month until 2023, without significance (p=0.567).

Regarding non-infectious lung conditions, we highlight that in group 7 (asthma), there was a reduction of 1.45% per month until 2020 (p=0.036) and an increase of 8.70% until 2023, without significance (p=0.098). In groups 8 (lung diseases) and 16 (skin infections), there was an increase of 1.95% (p<0.001) and a reduction of 0.38% per month (p<0.001), respectively. In group 19 (other causes), the variations were not statistically significant.

**Figure 2:**
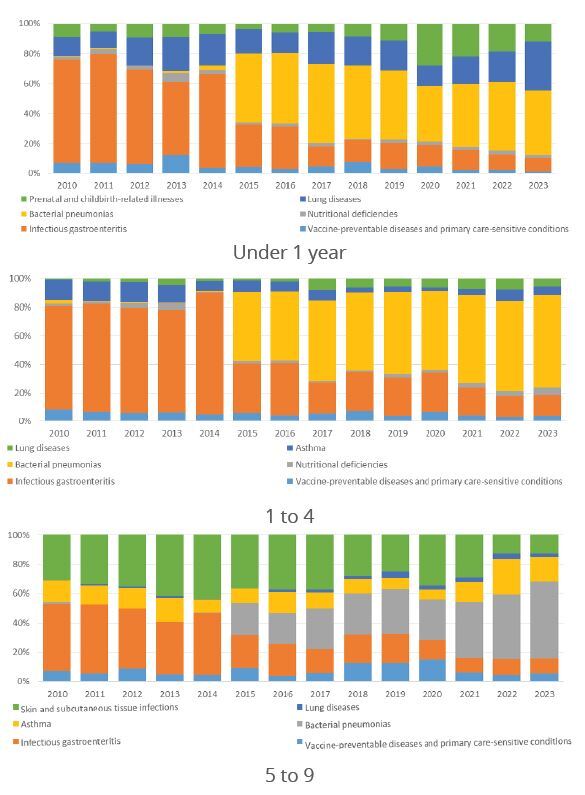
Proportion of HPCSC by groups in children under 1 year, 1 to 4 years and 5 to 9 years. Roraima, Brazil. 2010 to 2023.

HPCSC: hospitalizations for primary care-sensitive conditions; PHC: primary health care.

The composition of the main causes in each age group is shown in Figure 2. The growth in hospitalizations due to pneumonia is noticeable from 2015 onwards. In the joinpoint analysis by age group, similarity was observed in the behavior of hospitalizations, with a decrease in all segments at the beginning of the time series: children under 1 year old (-2.90%; 95%CI -7.75--0.64), 1 to 4 years old (-1.75%; 95%CI -4.09--0.51) and 5 to 9 years old (-0.79%; 95%CI -2.30--0.09). These segments were followed by an increase and a new decrease. In children under 1 year old, there was a monthly reduction of 9.90% in the segment from September 2019 to May 2020 (95%CI -30.32--0.97). In the age group from 1 to 4 years old, the reduction was 13.04% per month in the same period (95%CI -30.18--2.06). In the 5 to 9 age group, the most significant drop occurred at the beginning of the time series, in January.

## DISCUSSION

During the period analyzed, 35,684 HPCSC were recorded in children under 10 years of age in Roraima. In children under 1 year of age and children aged 1 to 4 years, recent periods showed a significant increase in rates, especially after 2020. Among the groups of causes, vaccine-preventable diseases and gastroenteritis had reductions in specific periods, while lung diseases and nutritional deficiencies showed constant growth. Bacterial pneumonias showed sharp variations, with significant reductions and peaks. These results show distinct patterns between age groups and groups of causes, indicating the need for targeted interventions. In the segmented analyses of the trend, we observed a very similar downward trend in the age groups in the third segment, which corresponds approximately to the end of 2019 until May 2020. This decline coincides with the first phase of the COVID-19 pandemic, which was from January to May 2020, a period that accumulated the highest number of hospitalizations due to COVID-19 in Brazil in children, mainly infants, which could explain the drop in HPCSC in this period^18^.

Among the groups of causes that showed an increasing trend, hospitalizations due to group 4 (nutritional deficiencies) stand out, as they showed constant growth in the period analyzed. This increase more recently may have been driven by the public health emergency of national importance in the Yanomami territory, which highlighted a high rate of nutritional deficit and food insecurity among this indigenous population^19^. Severe malnutrition is the main cause of air removal in the Yanomami territory, corresponding to 36% of all calls. Since 2015, the frequency of low weight in Yanomami children increased, reaching alarming levels. In the Surucucu base camp, for example, in 2022, malnutrition affected 71.4% of children^20^.

The increase in hospitalizations due to nutritional deficiencies that occurred since 2010 puts Roraima increasingly far from achieving the sustainable development goals (SDGs). There are 17 audacious goals that aim to overcome the greatest challenges that people around the world face. The second SDG is to end hunger, achieve food security and improve nutrition, and it seeks for nations to be able to guarantee access for all people, particularly the poor and people in vulnerable situations, including children, to safe, nutritious and sufficient food by 2030^21^.

In children under 1 year old, from 2010 to 2023, the largest representation of HPCSC in Roraima was in group 6, which corresponded to bacterial pneumonias. In Brazil, a study from 1998 to 2009 demonstrated that the main cause of HPCSC in children under 1 year old was infectious gastroenteritis (39.2%)^22^. This finding was similar to what we observed in Roraima until 2014. From that year on, bacterial pneumonias became the most common cause of HPCSC. In the state of Paraíba, in 2018, a study identified bacterial pneumonias as responsible for 48% of PCSC in children^23^.

The increase in hospitalizations due to pneumonias may be linked to the interruption of breastfeeding^23^ and also to the child’s own immunological condition, which is still developing. On the other hand, the increase in hospitalizations due to pneumonias in children under 1 year of age may indicate an improvement in clinical management and timely diagnosis of cases, since hospitalization is considered a treatment option for children under 2 months, since in this age group pneumonia should always be considered a serious disease^24^. In the age group of 1 to 4 years, pneumonia can be associated with several situations, the most important being malnutrition, young age and comorbidities, time spent in daycare, lack of breastfeeding, incomplete vaccination, socioeconomic variables and environmental variables^25^.

The reduction in hospitalizations for infectious gastroenteritis and complications was observed in Roraima, unlike the findings in other states, such as Tocantins, which had this cause of hospitalizations in children under 5 years of age between 2015 and 2020^26^. Gastroenteritis was also the main cause of hospitalizations for children under 5 years old in northeastern Brazil between 2003 and 2014^27^. Although gastroenteritis showed a decreasing trend in our findings, it still affects children throughout Brazil^26^, and reductions in these causes of hospitalizations indicate improvements in the health conditions of the population as a whole and demonstrate advances in PHC. Strategies such as vaccination of children against rotavirus and improvement of care can be linked to these reductions. Roraima has had a significant expansion in ESF coverage; In January 2021, the state’s PHC coverage was 62.6%, rising to 83.65% in July 2023^28^.

In the age group of 5 to 9, the number of hospitalizations due to skin and subcutaneous tissue infections is noteworthy, a finding that was also discussed in the state of Rondônia, where there was a significant increase in the number of children under 5 years of age^29^.

Considering the findings of this study, we believe it necessary to draw attention to the local specificities of the state’s health system. In 2016, pneumonia was already identified as a serious health problem in indigenous children. In that year, pneumonias accounted for 69.4% of HPCSC in Yanomami children in Roraima^30^. PHC for the indigenous population is provided by the Special Indigenous Health Districts^31^, which requires special attention to the analysis of hospitalizations in this specific population, taking into account the distinct organizational arrangements of indigenous PHC teams.

International immigration is a major public health challenge, capable of highlighting the weaknesses of health systems for the local population, highlighting existing difficulties^32^. Recently, the city government of the capital accredited two street clinic teams^33^, as a strategy to welcome the immigrant population, which largely lives in the public spaces of the capital, such as squares, streets and flowerbeds^6^. The clinic strategy can contribute to improving this situation, as it is capable of monitoring the movement of these populations in the city in real time, seeking equity and longitudinality of care, fundamental principles of the SUS^34^.

We believe it is important to mention that the humanitarian crisis among migrants and indigenous people is associated with other contextual effects. One of them is the pent-up demand for health services throughout the capital, largely by immigrants, including at the city’s children’s hospital, the only hospital care service for children in the state^16^. Although these are important aspects that have been considered in the current scenario of the state, it is important to note that other factors such as social inequality, education and income have influenced the behavior of HPCSC. Better municipal human development indexes associated with better living conditions were associated with lower HPCSC rates^35^, as was the social vulnerability index, which is a composite measure of 16 social indicators, such as income, access to work and urban infrastructure, among others. The social vulnerability index has already been cited as capable of exerting a greater influence on HPCSC than PHC coverage itself^36^.

The limitations of this study are those imposed on studies conducted with secondary data, given that these are generated by a system that depends on the quality of the information entered in the medical records, and also by the SIH, which only records those hospitalizations that were financically covered by SUS. It is also necessary to highlight the possibility of biases and overlapping explanatory factors in an ecological study such as this one. The use of the HPCSC indicator requires care in its interpretation, since, in addition to the quality of PHC, other factors, such as the socioeconomic conditions of the population and other organizational characteristics of the health system, can influence its outcome^35^ ^,^ ^36^.

The results indicated weaknesses in PHC, probably aggravated by the major migration crisis that the state is facing. Strategies are being sought to improve the quality of PHC, but they are still insufficient. PHC actions are needed to enhance care for users in an assertive manner, with intersectoral actions that are appropriate to specific needs, meeting the principles of equity and justice.
