# Supplementary material for: Time series of hospitalizations for primary care-sensitive conditions in children in the state of Roraima, Brazil, 2010 to 2023
Source: Rev Bras Epidemiol. 2025 Apr 7;28:e250016. doi: 10.1590/1980-549720250016 (PMC11980823; doi:10.1590/1980-549720250016)
Supplement: Translation

# Translation: Série temporal das internações por condições sensíveis à atenção primária em crianças no estado de Roraima, Brasil, 2010 a 2023

**DOI:** 10.1590/1980-549720250016.2

**Authors:** Ellen Vanuza Martins Bertelli, Gislayne Cristina Torreias de Carvalho, Raphael Mendonça Guimarães, Viviane Gomes Parreira Dutra

**Affiliations:** I Universidade Estácio de Sá - Rio de Janeiro (RJ), Brazil.; II Universidade Estadual de Roraima - Boa Vista (RR), Brazil.; III Fundação Oswaldo Cruz - Rio de Janeiro (RJ), Brazil.

**Keywords:** Atenção primária à saúde, Saúde da criança, Hospitalização, Epidemiologia

## Abstract

**Objetivo:**

Analisar a tendência das internações por condições sensíveis à atenção primária em crianças no estado de Roraima.

**Métodos:**

Estudo ecológico de série temporal, com dados secundários do Sistema de Informações Hospitalares sobre as internações ocorridas em menores de 10 anos no período de janeiro de 2010 a julho de 2023, analisados pelo método de regressão linear segmentada *joinpoint*.

**Resultados:**

A análise de tendência no período mostrou crescimento das internações em todas as faixas etárias. Houve redução dessas hospitalizações no grupo das doenças imunopreveníveis e condições sensíveis, no primeiro e no segundo segmento (mudança percentual mensal - MPC=-1.50; intervalo de confiança de 95% - IC95% -3,79-0,32; p=0,016; MPC=-1,91; IC95% -3,44--0,81 e p=0,007), das gastroenterites infecciosas, com queda constante em toda a série de 0,57% ao mês (IC95% -0,70--0,43 e p<0,001) e no grupo das infecções de pele e tecido subcutâneo (MPC=-0,38; IC95% -0,55--0,21 e p<0,001). A pneumonia bacteriana foi a principal causa de internação em todas as faixas etárias. O grupo das deficiências nutricionais apresentou crescimento uniforme das taxas de 1,56% ao mês (IC95% 0,27-2,80 e p=0,018). Houve queda das taxas no primeiro segmento em todas as idades: menor de 1 ano: -2,90% e p=0,019; 1 a 4 anos: -1,75% (p=0,011); e 5 a 9 anos: -0,79% (p=0,053), seguida por aumento e novamente queda.

**Conclusões:**

O cenário em Roraima é preocupante e necessita de intervenções urgentes, pois há indícios de fragilidades na atenção primária à saúde, provavelmente agravadas pela crise migratória. As estratégias buscadas ainda são insuficientes.

## INTRODUÇÃO

A atenção primária à saúde (APS) executa a coordenação dos cuidados em saúde^1^, o que significa promover a integração entre ações, serviços e profissionais, utilizando mecanismos e instrumentos específicos para o planejamento da assistência, definição de fluxos, troca de informações com os usuários e entre os profissionais. Dessa forma, a implementação de indicadores que monitoram e avaliam a efetividade da APS é substancial, pois tais indicadores fornecem uma medida objetiva do desempenho dos cuidados oferecidos aos usuários e permitem uma avaliação sistemática^1^ ^,^ ^2^.

Os indicadores de saúde da Região Norte brasileira evidenciam as fragilidades e iniquidades em saúde existentes ali^3^. Em 2022, o norte apresentou taxa de mortalidade infantil de 15,1/1.000 nascidos vivos, acima da taxa nacional, que foi de 12,6/1.000, revelando-se como a pior taxa entre as regiões. A mortalidade de menores de 5 anos foi de 18,5/1.000 nascidos vivos, enquanto no Brasil foi de 15/1.000. A razão de mortalidade materna na região também foi a maior do Brasil, alcançando 74,5/100 mil nascidos vivos^4^.

Entre os estados da região, Roraima possui a menor população, com 636.303 habitantes, e o menor número de municípios, apenas 15^5^. Embora seja o menor estado da federação, enfrenta há algum tempo desafios importantes para a organização dos serviços de saúde. Por um lado, há um intenso fluxo migratório cuja origem é predominantemente venezuelana, por causa de uma crise humanitária naquele país^6^. Além disso, há a intensificação de conflitos territoriais envolvendo populações indígenas, que têm sido isoladas e, consequentemente, vivenciado barreiras de acesso a qualquer serviço de saúde^7^. Um exemplo disso foi o surto de sarampo em 2018, que expôs a cobertura vacinal ineficiente do estado, que era inferior a 95%^8^.

O sistema de saúde do estado enfrenta desafios significativos, principalmente associado ao aumento da cobertura de saúde da família e à expansão de leitos hospitalares abaixo da média nacional^9^. De acordo com os dados de Giovanella et al.^10^, verificamos que a cobertura da estratégia saúde da família (ESF) é insuficiente, com índices abaixo da média nacional. A cobertura populacional pela ESF em Roraima é de 48,75%, conforme dados de maio de 2023 do e-Gestor AB do Ministério da Saúde, versus 62,1% de cobertura nacional. Em relação à densidade de médicos, o estado possui 1,82 médico por mil habitantes, o que equivale a 18,2 médicos para cada 10 mil habitantes, contra 24/10 mil no país como um todo, no entanto 97% desses profissionais estão na capital, Boa Vista, resultando em uma distribuição desigual. Quanto aos leitos hospitalares, Roraima apresentou crescimento de 24,1% na última década, passando de 1,73 para 2,15 leitos por mil habitantes entre 2012 e 2022. Isso corresponde a 21,5 leitos para cada 10 mil habitantes.

Uma das formas de monitorar indiretamente a qualidade dos serviços de atenção primária é por meio da utilização do indicador de internações por condições sensíveis à atenção primária (ICSAP). Esse indicador é comumente utilizado no mundo como avaliador indireto da qualidade da APS e tem demonstrado ser um parâmetro confiável de avaliação^11^. Quando a APS não oferece acesso suficiente nem adequado aos serviços de saúde, ocorre uma demanda excessiva por atendimentos nos níveis secundário e terciário, resultando no aumento das internações, dos custos e dos deslocamentos desnecessários^12^. No Brasil, a Portaria nº 221, de 17 de abril de 2008^13^, define as condições consideradas preveníveis pela APS.

Pesquisas nacionais sobre as ICSAP em crianças apontam perfis epidemiológicos distintos nos estados brasileiros. Na literatura recente, o único estudo que explora um estado do norte brasileiro é o de Santos et al.^14^, que analisou Rondônia no período de 2008 a 2019 e identificou que a maioria das ICSAP em crianças foi ocasionada por infecções de pele ou gastroenterites. Em relação às ICSAP em crianças no estado de Roraima, não foram encontrados estudos que abordassem especificamente essa temática. Diante disso, este estudo objetivou analisar a tendência das ICSAP em crianças no estado de Roraima.

## MÉTODOS

### Desenho e local de estudo

Estudo ecológico de série temporal realizado em Roraima. O estado está localizado na Região Norte do Brasil, é o menos populoso do país, com 636.303 habitantes, distribuídos em 15 municípios, segundo o censo de 2022^15^. A população é composta de uma significativa presença de comunidades indígenas, representando 14,1% dos habitantes, além de migrantes, especialmente da imigração venezuelana nos últimos anos. Geograficamente, Roraima é caracterizada por extensas florestas tropicais e comunidades isoladas, o que dificulta a logística e a prestação de serviços essenciais. Ainda, na história recente, o estado vivenciou fluxos migratórios e surtos de doenças^16^.

### Fonte de dados

Os dados utilizados foram captados do Sistema de Informações Hospitalares do Sistema Único de Saúde (SIH-SUS, https://datasus.saude.gov.br/transferencia-de-arquivos/) e extraídos do site do Departamento de Informática do SUS (DATASUS, https://datasus.saude.gov.br/), sobre as ICSAP ocorridas em menores de 10 anos entre janeiro de 2010 e julho de 2023, no estado de Roraima. O conjunto corresponde a todas as internações processadas no período e que constaram da autorização de internação hospitalar, no campo de justificativa de internação um dos códigos da Décima Revisão da Classificação Estatística Internacional de Doenças e Problemas Relacionados à Saúde que constam da Portaria nº 221, de 17 de abril de 2008^13^, conforme Quadro 1.

**Quadro 1:** Lista brasileira das condições sensíveis à atenção primária, Brasil, 2008.

Grupo	Nome	Classificação Internacional de Doenças, Décima Revisão (CID-10)
1	Doenças preveníveis por imunização e condições sensíveis	A37, A36, A33 a A35, B26, B06, B05, A95, B16, G00.0, A17.0, A19, A15.0 a A15.3, A16.0 a A16.2, A15.4 a A15.9, A16.3 a A16.9, A17.1 a A17.9, A18, I00 a I02, A51 a A53, B50 a B54, B77
2	Gastroenterites infecciosas e complicações	E86 e A00 a A09
3	Anemia	D50
4	Deficiências nutricionais	E40 a E46, E50 a E64
5	Infecções de ouvido, nariz e garganta	H66, J00, J01, J02, J03, J06, J31
6	Pneumonias bacterianas	J13, J14, J15.3, J15.4, J15.8, J15.9, J18.1
7	Asma	J45, J46
8	Doenças pulmonares	J20, J21, J40, J41, J42, J43, J47, J44
9	Hipertensão	I10, I11
10	Angina	I20
11	Insuficiência cardíaca	I50, J81
12	Doenças cerebrovasculares	I63 a I67; I69, G45 a G46
13	Diabetes mellitus	E10.0, E10.1, E11.0, E11.1, E12.0,E12.1; E13.0, E13.1; E14.0, E14.1, E10.2 a E10.8, E11.2 a E11.8; E12.2 a E12.8;E13.2 a E13.8; E14.2 a E14.8, E10.9, E11.9; E12.9, E13.9; E14.9
14	Eplepsias	G40, G41
15	Infecção no rim e trato urinário	N10, N11, N12, N30, N34, N39.0
16	Infecção da pele e tecido subcutâneo	A46, L01, L02, L03, L04, L08
17	Doença inflamatória dos órgãos pélvicos femininos	N70, N71, N72, N73, N75, N76
18	Úlceras gastrointestinais	K25 a K28, K92.0, K92.1, K92.2
19	Doenças relacionadas ao pré-natal e parto	O23, A50, P35.0

Fonte: Brasil^13^.

### Análise de dados

As internações foram analisadas por faixa etária: menores de 1 ano, de 1 a 4 anos e de 5 a 9 anos, e pelos grupos definidos na Portaria nº 221/2008. Para o cálculo dos coeficientes de internação, utilizamos como denominador as estimativas populacionais anuais do Instituto Brasileiro de Geografia e Estatística, disponíveis no site do DATASUS. As taxas de ICSAP foram calculadas separadamente para cada faixa etária, utilizando a Equação 1:

número de ICSAP ocorridas por ano e faixa etária / população do mesmo ano e na mesma faixa etária×10.000 (1)

Realizamos análise para grupos específicos de ICSAP, entre os 19 grupos existentes na Portaria nº 221/2008. É importante destacar que foram selecionados grupos cujos desfechos fossem coerentes com a saúde da criança. Os grupos selecionados foram:

- 1: Doenças preveníveis por imunização e condições sensíveis;
- 2: Gastroenterites infecciosas e complicações;
- 4: Deficiências nutricionais;
- 6: Pneumonias bacterianas;
- 7: Asma;
- 8: Doenças pulmonares;
- 16: Infecção da pele e tecido subcutâneo;
- 19: Doenças relacionadas ao pré-natal e parto.

A lista de grupos foi validada por um painel de especialista. Já nas análises por faixa etária, utilizamos todas as ICSAP ocorridas no período.

A taxa de ICSAP foi considerada como variável dependente, enquanto o ano, a variável independente. Foi realizada uma análise de regressão linear segmentada, por meio do método joinpoint, que permitiu identificar o incremento mensal, definido como a variação percentual mensal dos coeficientes de internação por CSAP. O método utilizado ajusta os dados da série de acordo com o menor número possível de joinpoints (0, ou seja, uma reta sem pontos de inflexão) e testa se a inclusão de mais joinpoints é estatisticamente significativa. Os testes de significância aplicados basearam-se no método de permutação de Monte Carlo e no cálculo da variação percentual mensal da razão, utilizando o logaritmo da razão^17^. Foi considerado o intervalo de confiança de 95% (IC95%). As análises foram realizadas no software Joinpoint Regression Program, versão 5.1.0.0.

O estudo seguiu as normas de pesquisa envolvendo seres humanos, conforme a Resolução nº 466/2012. Por se tratar de estudo realizado com dados secundários, cuja fonte é pública e cujo banco é não identificado, o estudo foi dispensado da apreciação por Comitê de Ética em Pesquisa com Seres Humanos.

## RESULTADOS

Durante o período estudado, foram registradas 35.684 ICSAP em crianças menores de 10 anos no estado de Roraima. Observou-se uma variação nas taxas ao longo dos anos e entre as diferentes faixas etárias. No geral, as taxas de ICSAP apresentaram comportamento dinâmico, com redução em determinados grupos de causas e segmentos temporais, seguido por crescimento em outros momentos (Figura 1).

**Figura 1:** Taxas de ICSAP/10 mil habitantes por faixa etária em crianças de 2010 a 2023. Roraima, Brasil.

ICSAP: internações por condições sensíveis a atenção primária.

A Tabela 1 apresenta a análise das taxas de ICSAP em diferentes faixas etárias, segmentadas por períodos temporais. Para cada segmento, são fornecidos os valores de mudança percentual mensal (MPC), os IC95% e os valores de significância estatística (valor p). Os resultados mostraram padrões distintos entre as faixas etárias e os períodos temporais analisados. Em menores de 1 ano e crianças de 1 a 4 anos, os períodos recentes apresentaram aumento significativo nas taxas de ICSAP, especialmente após 2020. Já na faixa etária de 5 a 9 anos, embora o crescimento tenha sido mais modesto, também foi registrado aumento significativo no período de abril de 2020 a julho de 2023.

**Tabela 1:** Estimativas de tendência temporal das taxas de ICSAP segundo pontos de corte obtidos por meio do joinpoint por faixa etária. Roraima, Brasil. Janeiro/2010 a agosto/2023.

Faixa etária (ano)	Segmento/Período		MPC	IC95%		Valor p
				LI	LS	
<1	Jan/2010	Mar/2012	-2,90	-7,75	-0,64	0,019
	Mar/2012	Set/2019	1,83	1,47	2,42	0,016
	Set/2019	Maio/2020	-9,90	-30,59	-0,80	0,027
	Maio/2020	Jul/2023	2,61	1,33	5,32	0,007
	Jan/2010	Jul/2023	0,62	0,34	0.93	0,005
1 a 4	Jan/2010	Dez/2012	-1,75	-4,09	-0,51	0,011
	Dez/2012	Nov/2019	1,10	0,77	1,77	0,003
	Nov/2019	Maio/2020	-13,04	-30,24	-1,82	0,009
	Maio/2020	Jul/2023	4,43	3,38	5,92	0,001
	Jan/2010	Jul/2023	0,68	0,45	0,90	<0,001
5 a 9	Jan/2010	Abr/2015	-0,79	-2,30	-0,11	0,053
	Abr/2015	Jan/2020	0,47	-11,62	18,39	0,154
	Jan/2020	Abr/2020	-18,05	-25,66	11,53	0,128
	Abr/2020	Jul/2023	3,69	2,24	5,69	0,033
	Jan/2010	Jul/2023	0,36	0,15	0,59	0,019

ICSAP: internações por condições sensíveis a atenção primária; MPC: mudança percentual mensal; IC95%: intervalo de confiança de 95%; LI: limite inferior; LS: limite superior.

Entre menores de 1 ano, as taxas apresentaram redução de 2,90% de janeiro de 2010 a março de 2012 (p = 0,019), seguida por aumento de 1,83% até setembro de 2019 (p = 0,016). Entre setembro de 2019 e maio de 2020, houve queda de 9,90% ao mês (p=0,027), com posterior aumento de 2,61% até julho de 2023 (p=0,007). No período total, registrou-se aumento geral de 0,62% ao mês (p=0,005).

Na faixa de 1 a 4 anos, houve redução de 1,75% ao mês de janeiro de 2010 a dezembro de 2012 (p = 0,011), seguida por aumento de 1,10% até novembro de 2019 (p=0,003). De novembro de 2019 a maio de 2020, registrou-se queda de -13,04% ao mês (p=0,009), com aumento posterior de 4,43% até julho de 2023 (p=0,001). No total, o aumento foi de 0,68% ao mês (p<0,001).

Entre 5 e 9 anos, observaram-se redução de 0,79% ao mês até abril de 2015 (p=0,053), aumento de 0,47% até janeiro de 2020 (não significativo) e queda de 18,05% até abril de 2020 (não significativa). De abril de 2020 a julho de 2023, houve aumento significativo de 3,69% ao mês (p=0,033), com aumento geral de 0,36% no período (p=0,019).

As ICSAP foram analisadas em diferentes grupos de causas (CSAP), revelando padrões distintos de MPC ao longo dos períodos (Tabela 2).

**Tabela 2:** Estimativas de tendência temporal das taxas de ICSAP segundo pontos de corte obtidos por meio do joinpoint por grupos de condições. Roraima, Brasil. Janeiro/2010 a julho/2023.

Grupo CSAP	Período/segmento		MPC	IC95%		Valor p
				LI	LS	
Grupo 1	Jan/2010	Out/2014	-1,50	-3,79	-0,32	0,016
	Out/2014	Jun/2018	2,83	1,22	50,94	0,009
	Jun/2018	Jul/2023	-1,91	-3,44	-0,81	0,007
Grupo 2	Jan/2010	Jul/2023	-0,57	-0,70	-0,43	<0,001
Grupo 4	Jan/2010	Jul/2023	1,56	0,27	2,80	0,018
Grupo 6	Jan/2010	Nov/2012	-22,84	-31,19	-14,67	<0,001
	Nov/2012	Ago/2015	53,15	38,83	86,15	<0,001
	Ago/2015	Jul/2023	0,65	-1,59	2,69	0,567
Grupo 7	Jan/2010	Out/2020	-1,45	-3,07	-0,31	0,036
	Out/2020	Jul/2023	8,70	-0,49	53,27	0,098
Grupo 8	Jan/2010	Jul/2023	1,95	1,17	2,76	<0,001
Grupo 16	Jan/2010	Jul/2023	-0,38	-0,55	-0,21	<0,001
Grupo 19	Jan/2010	Fev/2011	-26,76	-89,89	3,67	0,178
	Fev/2011	Jul/2023	3,44	-97,43	6,31	0,080

ICSAP: internações por condições sensíveis a atenção primária; CSAP: condições sensíveis a atenção primária; MPC: mudança percentual mensal; IC95%: intervalo de confiança de 95%; LI: limite inferior; LS: limite superior.

Com relação às doenças infecciosas ou imunopreveníveis, destacamos que no grupo 1 (doenças imunopreveníveis e condições sensíveis), houve redução de 1,50% ao mês entre janeiro de 2010 e outubro de 2014 (p=0,016), seguida por aumento de 2,83% ao mês até junho de 2018 (p=0,009) e nova redução de 1,91% ao mês até julho de 2023 (p=0,007). No grupo 2 (gastroenterites infecciosas), identificou-se redução constante de 0,57% ao mês entre 2010 e 2023 (p<0,001). Para o grupo 4 (deficiências nutricionais), houve aumento de 1,56% ao mês no mesmo período (p=0,018). No grupo 6 (pneumonias bacterianas), viram-se redução de 22,84% ao mês entre 2010 e 2012 (p<0,001), aumento de 53,15% ao mês até 2015 (p<0,001) e variação de 0,65% ao mês até 2023, sem significância (p=0,567).

No que diz respeito às condições pulmonares não infecciosas, ressaltamos que no grupo 7 (asma), houve redução de 1,45% ao mês até 2020 (p=0,036) e aumento de 8,70% até 2023, sem significância (p=0,098). Nos grupos 8 (doenças pulmonares) e 16 (infecções de pele), registraram-se aumento de 1,95% (p<0,001) e redução de 0,38% ao mês (p<0,001), respectivamente. No grupo 19 (outras causas), as variações não foram estatisticamente significativas.

A composição das principais causas em cada faixa etária é apresentada na Figura 2. O crescimento das internações por pneumonia é perceptível de 2015 em diante. Na análise de joinpoint por faixa etária, observou-se similaridade no comportamento das internações, com queda em todos os segmentos no início da série temporal: menores de 1 ano (-2,90%; IC95% -7,75--0,64), 1 a 4 anos (-1,75%; IC95% -4,09--0,51) e 5 a 9 anos (-0,79%; IC95% -2,30--0,09). Esses segmentos foram seguidos por aumento e nova queda. Em menores de 1 ano, houve redução mensal de 9,90% no segmento de setembro de 2019 a maio de 2020 (IC95% -30,32--0,97). Na faixa etária de 1 a 4 anos, a redução foi de 13,04% ao mês no mesmo período (IC95% -30,18--2,06). Já na faixa etária de 5 a 9 anos, a queda mais significativa ocorreu no início da série temporal, em janeiro.

**Figura 2:** Proporção de ICSAP por grupos em menores de 1 ano, 1 a 4 anos e 5 a 9 anos. Roraima, Brasil. 2010 a 2023.

ICSAP: internações por condições sensíveis a atenção primária; APS: atenção primária à saúde.

## DISCUSSÃO

Durante o período analisado, foram registradas 35.684 ICSAP em crianças menores de 10 anos em Roraima. Em menores de 1 ano e crianças de 1 a 4 anos, os períodos recentes apresentaram aumento significativo nas taxas, especialmente após 2020. Entre os grupos de causas, doenças imunopreveníveis e gastroenterites tiveram reduções em períodos específicos, enquanto doenças pulmonares e deficiências nutricionais mostraram crescimento constante. Pneumonias bacterianas apresentaram variações acentuadas, com reduções e picos significativos. Esses resultados evidenciam padrões distintos entre as faixas etárias e grupos de causas, indicando necessidade de intervenções direcionadas.

Nas análises segmentadas da tendência visualizamos um comportamento de queda muito similar nas faixas etárias no terceiro segmento, que corresponde a aproximadamente o fim de 2019 até maio de 2020. Essa queda coincide com a primeira fase da pandemia da COVID-19, que foi de janeiro a maio de 2020, período que acumulou os maiores números de internações por COVID-19 no Brasil em crianças, principalmente lactentes, o que poderia explicar a queda das ICSAP nesse período^18^.

Entre os grupos de causas que tiveram tendência de aumento, chamam a atenção as internações pelo grupo 4 (deficiências nutricionais), que apresentaram constante crescimento no período analisado. Esse aumento mais recentemente pode ter sido impulsionado pela emergência em saúde pública de importância nacional no território Yanomami, que evidenciou alto índice de déficit nutricional e insegurança alimentar entre essa população indígena^19^. A desnutrição grave é a maior causa de remoção aérea em território Yanomami, correspondendo a 36% de todos os chamados. Desde o ano de 2015, a frequência de baixo peso em crianças Yanomamis tem aumentado, até alcançar valores alarmantes. No polo-base Surucucu, por exemplo, em 2022 a desnutrição atingia 71,4% das crianças^20^.

O aumento das internações por deficiências nutricionais que vêm ocorrendo desde 2010 coloca Roraima cada vez mais distante de atingir os objetivos do desenvolvimento sustentável (ODS). São 17 objetivos audaciosos que visam superar os maiores desafios que pessoas de todo o mundo enfrentam. O segundo ODS é acabar com a fome, alcançar a segurança alimentar e melhorar da nutrição e busca que as nações consigam até 2030 garantir o acesso de todas as pessoas, particularmente os pobres e pessoas em situações vulneráveis, incluindo crianças, à alimentação segura, nutritiva e suficiente^21^.

Em menores de 1 ano, no período de 2010 a 2023, a maior representação de ICSAP no estado de Roraima foi do grupo 6, que corresponde às pneumonias bacterianas. No Brasil, um estudo de 1998 a 2009 demonstrou como a principal causa de ICSAP em menores de 1 ano é a gastroenterite infecciosa (39,2%)^22^. Esse achado foi semelhante ao que observamos em Roraima até o ano de 2014. Desse ano em diante, a pneumonia bacteriana passou a ter a maior representação entre as ICSAP. No estado da Paraíba, no ano de 2018, estudo identificou a pneumonia bacteriana como responsável por 48% das ICSAP em crianças^23^.

O aumento das internações por pneumonias pode estar atrelado à interrupção do aleitamento materno^23^ e também à própria condição imunológica da criança, que ainda se encontra em formação. Em contrapartida, o aumento das internações por pneumonia em menores de 1 ano pode indicar melhoria no manejo clínico e no diagnóstico oportuno dos casos, já que a internação é considerada uma opção de tratamento para menores de 2 meses, visto que nessa faixa etária a pneumonia deve sempre ser considerada uma doença grave^24^. Na faixa etária de 1 a 4 anos a pneumonia pode ser associada a diversas situações, sendo as mais importantes a desnutrição, a baixa idade e as comorbidades, permanência em creche, ausência de aleitamento materno, vacinação incompleta, variáveis socioeconômicas e variáveis ambientais^25^.

A redução das internações por gastroenterites infecciosas e complicações foi observado em Roraima, diferentemente dos achados em outros estados, como em Tocantins, que no período de 2015 a 2020 tinha essa causa de internações em crianças menores de 5 anos como a principal^26^. As gastroenterites também foram as principais causas de ICSAP em menores de 5 anos no nordeste brasileiro entre os anos de 2003 e 2014^27^. Apesar de as gastroenterites apresentarem tendência decrescente em nossos achados, ainda afetam as crianças de todo o Brasil^26^, e reduções nessas causas de internações remetem a melhorias nas condições de saúde da população como um todo e evidenciam avanços da APS. Estratégias como vacinação de crianças contra o rotavírus e qualificação do cuidado podem ser atreladas a essas reduções. Roraima teve significativa expansão na cobertura da ESF; em janeiro de 2021, a cobertura de APS do estado era de 62,6%, avançando para 83,65% em julho de 2023^28^.

Na faixa etária de 5 a 9 anos, chama a atenção o aparecimento das internações por infecções de pele e tecido subcutâneo, achado que também foi discutido no estado de Rondônia, onde teve representatividade e crescimento em menores de 5 anos^29^.

Considerando os achados deste estudo, consideramos necessário chamar a atenção para as especificidades locais do sistema de saúde do estado. Já no ano de 2016 se identificava em crianças indígenas a pneumonia como um grave problema de saúde. Nesse ano, a pneumonia correspondia a 69,4% das ICSAP em crianças Yanomami internadas em Roraima^30^. A APS à população indígena é prestada pelos Distritos Sanitários Especiais Indígena^31^, o que requer atenção especial às análises das internações nessa população específica, levando-se em conta os arranjos organizacionais distintos das equipes de APS indígenas.

A imigração internacional é um grande desafio de saúde pública, capaz de evidenciar as fragilidades dos sistemas de saúde para a população local, destacando dificuldades existentes^32^. Recentemente a gestão municipal da capital credenciou duas equipes de consultório na rua^33^, como estratégia de acolhimento para a população imigrante, que se aloja, em grande parte, nos espaços públicos da capital, como praças, ruas e canteiros^6^. A estratégia do consultório pode contribuir com a melhoria dessa situação, visto ser capaz de acompanhar em tempo real o deslocamento dessas populações na cidade, buscando equidade e longitudinalidade do cuidado, princípios fundamentais do SUS^34^.

Consideramos importante mencionar que a crise humanitária entre migrantes e entre os indígenas está associada a outros efeitos contextuais. Um deles é o represamento de demanda aos serviços de saúde por toda a capital, em grande parte por imigrantes, incluindo no hospital infantil da cidade, único serviço de atenção hospitalar para crianças do estado^16^. Apesar de serem importantes aspectos que têm sido considerados no cenário atual do estado, é preciso que se observe que outros fatores como desigualdade social, escolaridade e renda têm influenciado no comportamento das ICSAP. Melhores índice de desenvolvimento humano municipal associado a melhores condições de vida foram associados a quedas das taxas de ICSAP^35^, assim como o índice de vulnerabilidade social (IVS), que é uma medida composta de 16 indicadores sociais, como renda, acesso ao trabalho, infraestrutura urbana, entre outros. O IVS já foi citado como capaz de exercer maior influência nas ICSAP do que a cobertura da APS em si^36^.

As limitações deste estudo são aquelas impostas a trabalhos realizados com dados secundários, haja vista que estes são gerados por um sistema que depende da qualidade da informação preenchida nos prontuários, e ainda do SIH, que só registra aquelas hospitalizações que foram financiadas pelo SUS. Também há que se evidenciar a possibilidade de vieses e imbricados fatores explicativos em um estudo ecológico como este. O uso do indicador ICSAP requer cuidado com sua interpretação, pois, além da qualidade da APS, outros fatores, como as condições socioeconômicas da população e outras características organizacionais do sistema de saúde, podem influenciar em seu desfecho^35^ ^,^ ^36^.

Os resultados indicaram fragilidades na APS, provavelmente agravadas pela grande crise migratória que o estado enfrenta. Estratégias estão sendo buscadas para melhoria da qualidade da APS, porém ainda são insuficientes. Fazem-se necessárias ações da APS que potencializem o cuidado aos usuários de forma assertiva, com ações intersetoriais e adequadas às necessidades específicas atendendo aos princípios de equidade e justiça.
